# Available Evidence and Ongoing Clinical Trials of Remdesivir: Could It Be a Promising Therapeutic Option for COVID-19?

**DOI:** 10.3389/fphar.2020.00791

**Published:** 2020-05-26

**Authors:** Mekonnen Sisay

**Affiliations:** Department of Pharmacology and Toxicology, School of Pharmacy, College of Health and Medical Sciences, Haramaya University, Harar, Ethiopia

**Keywords:** SARS-CoV-2, COVID-19, remdesivir, RdRp, GS-5734

## Abstract

The novel coronavirus strain, severe acute respiratory syndrome coronavirus-2, the causative agent of COVID-19 emerged in Wuhan, China, in December 2019 and is skyrocketing throughout the globe and become a global public health emergency. Despite promising preventive measures being taken, there is no vaccine or drug therapy officially approved to prevent or treat the infection. Everybody is waiting the findings of ongoing clinical trials in various chemical and biological products. This review is specifically aimed to summarize the available evidence and ongoing clinical trials of remdesivir as a potential therapeutic option for COVID-19. Remdesivir is an investigational drug having broad spectrum antiviral activity with its target RNA dependent RNA polymerase. It has not yet been officially approved for Ebola and Coronaviruses. Several studies showed that remdesivir had promising *in vitro* and *in vivo* antiviral activities against SARS-CoV-1 and MERS-CoV strains. On the top of this, it exhibited a promising *in vitro* activity against SARS-CoV-2 strains though there are no published studies that substantiate its activity *in vivo* until the time of this review. There are few phase 3 randomized double-blind placebo controlled trials on the way to investigate the safety and efficacy of remdesivir. Of which, one completed double blind, placebo controlled trial showed that remdesivir showed faster time to clinical improvement in severe COVID-19 patients compared to placebo though not found statistically significant. In addition, two phase 3 randomized open label clinical trials coordinated by Gilead Sciences are being conducted. In addition, WHO Solidarity trial and INSERM DisCoVeRy trials (randomized open labels) were launched recently.

## Background

Coronaviruses, belonging to the family Coronaviridae, are positive-sense enveloped RNA viruses that cause infections in humans (Weiss and Leibowitz, 2011; Lim et al., 2016). The family includes four genera (Alphacoronavirus, Betacoronavirus, Deltacoronavirus, and Gammacoronavirus). The genus Betacoronavirus includes severe acute respiratory syndrome coronavirus (SARS-CoV) and Middle East respiratory syndrome coronavirus (MERS-CoV). Historically, these coronaviruses had got great clinical importance in infecting humans (Kuiken et al., 2003). At present, the novel coronavirus strain, the SARS-CoV-2, the causative agent of COVID-19 emerged in Wuhan, China, in December 2019 (Zhu et al., 2020). Since then, the number of cases and deaths related to this virus have been skyrocketing throughout the world.

As per the World Health Organization (WHO) report, the total number of cases and deaths outside China has overtaken the total number of cases in China (WHO, 2020). WHO has declared COVID-19 worldwide pandemic and global public health emergency with Europe and lately Unites States of America became new epicenters. WHO has recommended several preventive measures including laboratory tests for any suspected cases, quarantining suspects, applying physical distancing, frequent hand washing, and using hand and surface sanitizers to help contain further spread of the pandemic (WHO, 2020). Despite such preventive strategies, there is no vaccine or drug therapy officially approved for prophylaxis or treatment of COVID-19. At present, there are several classes of drugs undergoing clinical trials including RNA polymerase inhibitors (remdesivir and favipiravir), protease inhibitors (lopinavir/ritonavir), aminoquinolines (chloroquine and its hydroxyl derivative), anti-inflammatory agents (corticosteroids, and xiyanping injection), angiotensin converting enzyme type 2 blockers, convalescent plasma, viral RNA antisense technologies, monoclonal antibodies, and Chinese traditional medicines (http://www.chictr.org.cn/index.aspx and https://clinicaltrials.gov/ct2/home).

## Overview of Other Frontline Antiviral Agents Under Extensive Clinical Investigation

Among the above-mentioned classes of antiviral counterparts, aminoquinolines (chloroquine and its hydroxyl derivative) and protease inhibitors (primarily lopinavir/ritonavir) have taken the largest share of clinical trials since the SARS-CoV-2 outbreak. However, their clinical benefit has become full of controversies according to the various research findings.

With regard to the aminoquinolines and their role in COVID-19 therapy, in an open-label non-randomized clinical trial, Gautret et al. reported 100% viral clearance in nasopharyngeal swabs with combination of hydroxychloroquine and azithromycin, 57.1% in hydroxychloroquine group, and 12.5% in standard of care group in cohort of 6 patients after 5 to 6 days follow-up (Gautret et al., 2020). In addition, in study conducted in 62 patients in China, the use of hydroxychloroquine could significantly shorten the time to clinical recovery and promote the absorption of pneumonia in randomized open label clinical trial (Chen Z. et al., 2020). In contrary to this, a recent study from China in individuals with COVID-19 found no difference in the rate of virologic clearance at 7 days with or without 5 days of hydroxychloroquine, and no difference in clinical outcomes (Chen J. et al., 2020) indicating the absence of evidence of a strong antiviral activity (rapid viral clearance) or clinical benefit of this combination for severe COVID-19 patients. Another randomized open label clinical trial posted on MedRxiv (preprint) reported that the overall 28-day negative conversion rate and symptoms alleviation rate in hydroxychloroquine plus standard of care group was not different from standard of care group (Tang et al., 2020). Apart from this, the safety issues of aminoquinolines should also be emphasized. The cardiovascular and retinal toxicities may also limit the usefulness of these agents if there is hope from ongoing randomized, blinded and placebo controlled trials. For example, the hydroxychloroquine and azithromycin have shown to prolong QT-interval resulting torsades de pointes (a form of polymorphic ventricular tachycardia) and sudden cardiac death (Chorin et al., 2020).

A randomized, open label clinical trial conducted on protease inhibitors (lopinavir/ritonavir) indicated that treatment with lopinavir/ritonavir did not show statistically significant difference in the time to clinical improvement and mortality censored at day 28 though a secondary outcomes measures were found promising in lopinavir/ritonavir group (Cao et al., 2020). This trial was initiated in severe COVID-19 patients lately and lacks blinding and well established placebo.

Remdesivir is one of the frontline medications being used as expanded access and is under extensive clinical investigation. Hereafter, this review aims to address the viral polymerase inhibitor remdesivir as a potential therapeutic option for COVID-19.

## Remdesivir: Chemistry and Mechanism of Action

**Figure 1:**
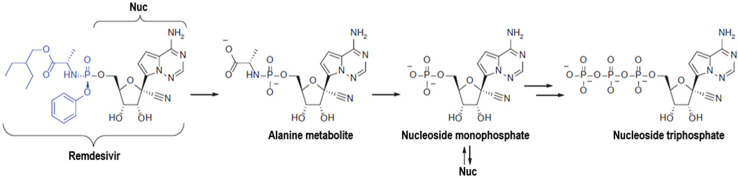
Structure and metabolic conversion of remdesivir.

Remdesivir (GS-5734™) is a phosphoramidate prodrug of a Pyrrolo[2,1-*f]*[triazin-4-amino] adenine **C***-*nucleoside having broad spectrum antiviral activity (**Figure 1**). Remdesivir is metabolized into its active form, GS-441524, that interferes with viral RNA dependent RNA polymerase (RdRp) enzyme thereby it evades proofreading by viral exonuclease, and arrests RNA synthesis. This drug has shown potent inhibitory activity against RdRp with intact proof reading and with low level of resistance to target mutations (Agostini et al., 2018). It is supported in the study conducted by Gordon et al. who demonstrated RdRp is, indeed, the target of remdesivir in MERS-CoV strains (Gordon et al., 2020). Studies based on the molecular dynamics simulation and free energy perturbation methods clearly indicated SARS-CoV-2 RdRp as a target of remdesivir (Zhang and Zhou, 2020). Though, it has not been officially approved for Ebola and Coronaviruses yet (Siegel et al., 2017), Gilead Sciences is working closely with organizations and health authorities to respond to the COVID-19 outbreak through synthesizing and providing this investigational drug (Gilead sciences, 2020a).

## Evidence on Its *in Vitro* Activity

Agostini et al. demonstrated that remdesivir can potently inhibit coronaviruses such as SARS-CoV-1 and MERS-CoV *in vitro* (Agostini et al., 2018). Remdesivir can inhibit SARS-CoV-1 and MERS-CoV replication in several *in vitro* systems, including primary human airway epithelial cell cultures (Sheahan et al., 2017). In research conducted by Sheahan et al, remdesivir showed superior antiviral activity to lopinavir/ritonavir against MERS-COV *in vitro* (Sheahan et al., 2020). In MERS-COV nonstructural proteins (nsp5, nsp7, nsp8, and nsp12) of insect cell lines, remdesivir showed potent inhibitory activity against nsp12(RdRp) *in vitro* (Gordon et al., 2020). Yethindra et al. demonstrated that remdesivir showed strong inhibition against SARS-CoV and MERS-CoV in human air way epithelial cells, at early stages in replication process *via* inhibiting viral RNA synthesis (Yethindra, 2020). On the top of these, remdesivir has shown promising results in clinical control of SARS-CoV-2 pneumonia *in-vitro* in human liver cancer cell lines (Wang M. et al., 2020). Beyond Beta-CoVs, remdesivir has shown potent inhibition of human endemic and zoonotic Delta-CoVs with highly divergent RdRp in human hematoma (huh7) cell lines (Brown et al., 2019).

## Evidence on *in Vivo* Antiviral Activity

In a mouse model of SARS-CoV-1, prophylactic and therapeutic (at early stage) administration of remdesivir significantly reduced pulmonary viral load and improved respiratory function and other clinical signs of the disease (Sheahan et al., 2017). Likewise, both prophylactic and therapeutic remdesivir has shown improvement on the pulmonary function and reduced lung viral loads and severe lung pathology in MERS-COV strains in mice model (Sheahan et al., 2020). In the rhesus macaque model of MERS-CoV infection, remdesivir reduced virus replication, the severity of the disease, and lung damage when administered in animals infected with MERS-CoV (de Wit et al., 2020). Despite having an *in-vitro* antiviral activity against SARS-CoV-2, there are no published studies justifying the activity of remdesivir in animal models of SARS-CoV-2 *in vivo* until the time of this review.

## Case Report

According to the paper published at the New England Journal of Medicine on 05 March, 2020, it had been suggested that remdesivir might be a potential therapeutic option for the therapy of COVID-19 patients. In the report, remdisivir intravenous infusion (compassionate use) was started on day 7 in COVID-19 patient. During the treatemnt, no adverse events were observed in association with the IV infusion. The patient's clinical condition improved. The bilateral lower-lobe rales apeared initially were no longer present. His appetite improved, became afebrile and asymptomatic except intermittent dry cough and rhinorrhea (Holshue et al., 2020). However, this is a single patient report and is too infant to conclude its efficacy and disentangle the true effect size of this drug because of the chance of recovery from this disease without treatment(s). Hence, it is imperative to have adequate, well controlled, randomized, and blinded clinical trials in large cohorts of patients to justify its clinical utility in real settings.

## Ongoing Clinical Trials and Future Prospects

As summarized in **Table 1**, Gilead Sciences has initiated two phase 3 randomized, open label clinical trials comprising approximately 1,000 COVID-19 patients. In the first trial, 400 patients with severe COVID-19 cases were enrolled to evaluate the safety and efficacy of remdesivir on 5 (Arm 1) and 10 days (Arm 2) regimens with standard of care in both arms without comparator (Gilead-Sciences, 2020b). In the second trial, 600 patients with moderate COVID-19 cases were enrolled to evaluate the safety and efficacy of the same dosage regimen of remdesivir in addition to the standard of care and with standrad of care alone as active comparator (Gilead-Sciences, 2020c).

**Table 1:** Ongoing clinical trials registered under United States National Library of Medicine clinical trials registry and addressing the safety and efficacy of remdesivir (GS-5734™) as a potential therapeutic option for COVID 19.

Clinical trial identifier	Study design	Estimated enrollment	Phase of the study	Conditions	Interventions		Primary outcome measures	Recruitment status
					Experimental arm	Comparator (control) arm		
NCT04292899 (Gilead-Sciences, 2020b)	Randomized, open label clinical trial	400	Phase 3	Severe COVID 19	**Arm 1:** Remdesivir IV infusion for 5 Days + standardized care **Arm 2**: Remdesivir IV infusion 10 Days	**Active:** No placebo	Composite outcome measure (proportion of participants with normalization of fever and oxygen saturation through day 14)	Recruiting
NCT04292730 (Gilead-Sciences, 2020c)	Randomized, open label clinical trial	600	Phase 3	Moderate COVID 19	**Arm 1:** Remdesivir IV infusion for 5 Days + standardized care **Arm 2:** Remdesivir IV infusion 10 Days	**Arm 3:** Active (Standard of Care)	Proportion of participants discharged by day 14	Recruiting
NCT04252664 (Cao, 2020a)	Randomized, Double-blind, Placebo-controlled clinical trial	308	Phase 3	Mild and Moderate COVID 19	**Arm 1:** Remdesivir IV for 10 Days (200 mg loading dose for day 1 followed by 100 mg IV for 9 days)	**Arm 2**: Placebo that match remdesivir in dose and duration	Time to clinical recovery (TTCR) Upto 28 days	Recruiting
NCT04257656 (Cao, 2020b)	Randomized, Double-blind, Placebo-controlled clinical trial	453	Phase 3	Severe COVID 19	**Arm 1:** Remdesivir IV for 10 Days (200 mg loading dose for day 1 followed by 100 mg IV for 9 days)	**Arm 2:** Placebo that match remdesivir in dose and duration	Time to Clinical Improvement (TTCI) [Censored at Day 28]	Completed
NCT04280705 (NIAID, 2020)	Adaptive, Randomized, double Blind Controlled Trial	394 (1:1)	Phase 3	Hospitalized patients with COVID 19 (no specific severity)	**Arm 1:** 200 mg RDV IV for day 1 followed by 100 mg IV QD for 9 days	**Arm 2**: Placebo that match remdesivir in dose and duration	Percentage of subjects reporting each severity rating on the 7-point ordinal scale within 15 days	Recruiting
NCT04302766 (US-AMRDC, 2020)	Expanded access	General (Intermediate-size Population)	NA	Any COVID 19 case	Not stated	Not stated	NA	Available
NCT04315948 **(DisCoVeRy trial**) (INSERM, 2020)	Adaptive, Randomized, Open label clinical Trial	3200	Phase 3	COVID-19 in hospitalized adults	**Arm 1:** 200 mg RDV IV for day 1 followed by 100 mg IV QD for 9 days **Arm 2:** Lopinavir/ritonavir (400 mg/100 mg) tablet BID for 14 days **Arm 3:** Lopinavir/ritonavir (400 mg/100 mg) tablet BID for 14 days plus Interferon ß-1a 44 ug subcutaneously in total of three doses (Day 1, 3 and 6)	**Arm 4:** (Active) Standard of care	Percentage of subjects reporting each severity rating on a 7-point ordinal scale within 15 days	Not yet recruiting

With the coordination of China-Japan Friendship Hospital, two phase 3 randomized, double blind, placebo controlled clinical trials were intiated in China to evaluate the safety and efficacy of remdesivir paralleled to palcebo therapy.The first phase 3 trial has already involved 308 hospitalized adult patients with mild to moderate cases of COVID-19 (Cao, 2020a). The patients were randomized to intervention arms of either remdesivir 10 days regimen or placebo that matched to remdesivir. The primary end point of this trial is set to determine the time to clinical recovery (TTCR) within 28 days. Another phase 3 randomized, double-blind, placebo-controlled trial is evaluating the safety and efficacy of 10 days of remdesivir regimen in 453 hospitalized adult patients with severe COVID-19 compared to placebo matched to remdesivir in dose and duration. The primary outcome measure in this trial is the time to clinical improvement (TTCI) censored at day 28 (Cao, 2020b) (**Table 1**). At the time of this revision, the finding of the second trial was published on Lancet. Though not statistically significant, patients receiving remdesivir had a numerically faster time to clinical improvement than those receiving placebo among patients with symptom duration of 10 days or less (Hazard ratio 1.52 [0.95–2.43]). In addition, remdesivir did not show statistically significant clinical benefits (Wang Y. et al., 2020). However, the numerical reduction in time to clinical improvement in those treated earlier requires confirmation in the larger cohort of patients and in the remaining trials

With the coordination of the U.S. National Institute of Allergy and Infectious Diseases (NIAID), Another phase 3 adaptive, randomized, double blind, placebo controlled clinical trial enrolled 394 hospitalized patients with COVID-19 to assess the safety and efficacy of remdesivir. In this trial, patients were randomized to either placebo or 10 days therapy with remdesivir (200 mg remdesivir IV loading dose for day 1 followed by 100 mg IV daily maintained for 9 days, total of 10 days therapy). The primary indicator in this trial is the percentage of patients reporting each severity rating on the 7-point ordinal scale within 15 days (NIAID, 2020) (**Table 1**).

With the sponsor and coordination of U.S. Army Medical Research and Development Command (AMRDC), remdesivir is also provided as expanded access (compassionate use) (US-AMRDC, 2020) through emergency investigational new drug applications. The term expanded access (compassionate use) is a potential pathway in which a patient with an immediately life-threatening condition or disease gain access to an investigational medical product for treatment of patients outside of clinical trials when no comparable or satisfactory alternative therapy options are available (FDA, 2020). To this end, remdesivir synthesized and developed by Gilead Sciences is readily available for compassionate use for COVID-19 patients (Coppock, 2020) (**Table 1**). Latest observational study published on The New England Journal of Medicine revealed that severe Covid-19 patients treated with compassionate-use of remdesivir showed clinical improvement in 68% cases (Grein et al., 2020).

On the top of this, WHO has initiated Solidarity trial at global level to evaluate the safety and efficacy of remdesivir in one of the interventional arms (Branswell, 2020). Likewise, National Institute of Health and Medical Research (INSERM) of France has planned to initiate DisCoVeRy trial (INSERM, 2020). The trial protocol has already been registered on 20 March, 2020 and available at https://clinicaltrials.gov/ct2/show/NCT04315948. The recruiting of participants has not been started but estimated to enroll 3,200 patients in 4 arms in which remdesivir alone is to be provided in usual dosage regimen in one of the interventional arms.

## Conclusion

Despite the promising effects of remdesivir against previous beta-coronaviruses as well as the current novel coronaviruses *in vitro*, there is no published *in vivo* study that substantiates the *in vitro* activities against this global public health threat. A case report and observational studies are not sufficient to generate evidenced-based medicine on the clinical use of remdesivir for this pandemic. A double blind, placebo controlled randomized clinical trial showed that remdesivir did not have statistically significant clinical benefit in reducing the time to clinical improvement in severe COVID-19 patients compared to placebo. Though remdesivir is readily available for compassionate use in many countries, it is imperative to wait the remaining ongoing clinical trials to justify its clinical utility on larger cohort of COVID-19 patients.

## Author Contributions

The author confirms being the sole contributor of this work and has approved it for publication.

## Conflict of Interest

The author declares that the research was conducted in the absence of any commercial or financial relationships that could be construed as a potential conflict of interest.

## References

[B1] Agostini M. L. Andres E. L. Sims A. C. Graham R. L. Sheahan T. P. Lu X. (2018). Coronavirus susceptibility to the antiviral remdesivir (GS-5734) is mediated by the viral polymerase and the proofreading exoribonuclease. MBio 9, e`1–00218. 10.1128/mBio.00221-18 PMC5844999 29511076

[B2] Branswell H. (2020). WHO to launch multinational trial to jumpstart search for coronavirus drugs (World Health Organization). Available at: https://www.statnews.com/2020/03/18/who-to-launch-multinational-trial-to-jumpstart-search-for-coronavirus-drugs/ Accessed on 20 March, 2020.

[B3] Brown A. J. Won J. J. Graham R. L. Dinnon III K. H. Sims A. C. Feng J. Y. (2019). Broad spectrum antiviral remdesivir inhibits human endemic and zoonotic deltacoronaviruses with a highly divergent RNA dependent RNA polymerase. Antiviral Res. 169, 104541. 10.1016/j.antiviral.2019.104541 31233808 PMC6699884

[B4] Cao B. Wang Y. Wen D. Liu W. Jingli W. Fan G. (2020). A Trial of Lopinavir–Ritonavir in Adults Hospitalized with Severe Covid-19. New Engl. J. Med. 10.1056/NEJMoa2001282. 2020. PMC7121492 32187464

[B5] Cao B. (2020a). Mild/Moderate 2019-nCoV Remdesivir RCT, Available at: https://clinicaltrials.gov/ct2/show/NCT04252664 Accessed on 20, March, 2020.

[B6] Cao B. (2020b). Severe 2019-nCoV Remdesivir RCT, avaialble at: https://clinicaltrials.gov/ct2/show/NCT04257656 Accessed on 20 March, 2020.

[B7] Chen J. Liu D. Lui L. Liu P. Xu Q. Xia L. (2020). A pilot study of hydroxychloroquine in treatment of patients with common coronavirus disease-19 COVID-19). J. Zhejiang Univ. Sci. 49, 215–219. 10.3785/j.issn.1008-9292.2020.03.03 PMC8800713 32391667

[B8] Chen Z. Hu J. Zhang Z. Jiang S. Han S. Yan D. (2020). Efficacy of hydroxychloroquine in patients with COVID-19: results of a randomized clinical trial. medRxiv. 10.1101/2020.03.22.20040758

[B9] Chorin E. Dai M. Shulman E. Wadhwani L. Bar-Cohen R. Barbhaiya C. (2020). The QT interval in patients with COVID-19 treated with hydroxychloroquine and azithromycin. Nat. Med. 10.1038/s41591-020-0888-2 32488217

[B10] Coppock K. (2020). FDA Announces Two Drugs Approved for ‘Compassionate Use' in Treating COVID-19. (Pharmacy Times). Available at: https://www.pharmacytimes.com/news/fda-announces-two-drugs-approved-for-compassionate-use-in-treating-covid-19 Accessed on 20 March, 2020.

[B11] de Wit E. Feldmann F. Cronin J. Jordan R. Okumura A. Thomas T. (2020). Prophylactic and therapeutic remdesivir (GS-5734) treatment in the rhesus macaque model of MERS-CoV infection. Proc. Natl. Acad. Sci. 117, 6771–6776. 10.1073/pnas.1922083117 PNAS 32054787 10.1073/pnas.1922083117 PMC7104368

[B12] FDA (2020). United States Food and Drug Administration, Expanded Access. (U.S. FDA). Available at: https://www.fda.gov/news-events/public-health-focus/expanded-access Accessed on 20 March, 2020.

[B13] Gautret P. Lagier J. C. Parola P. Hoang V. T. Meddeb L. Mailhe M. (2020). Hydroxychloroquine and azithromycin as atreatment of COVID-19: results of an open-label non-randomised clinical trial. Int. J. Antimicrob. Agents. 10.1016/j.ijantimicag.2020.105949 PMC7102549 32205204

[B14] Gilead sciences (2020a). Gilead Sciences Update On The Company"s Ongoing Response To COVID-19. (GILEAD). Available at: https://www.gilead.com/purpose/advancing-global-health/covid-19 Accessed on 20 March, 2020.

[B15] Gilead-Sciences (2020b). Study to Evaluate the Safety and Antiviral Activity of Remdesivir (GS-5734™) in Participants With Severe Coronavirus Disease (COVID-19), Available at: https://clinicaltrials.gov/ct2/show/NCT04292899 Accessed on 20 March, 2020.

[B16] Gilead-Sciences (2020c). Study to Evaluate the Safety and Antiviral Activity of Remdesivir (GS-5734™) in Participants With Moderate Coronavirus Disease (COVID-19) Compared to Standard of Care Treatment, Available at: https://clinicaltrials.gov/ct2/show/NCT04292730 Accessed on 20 March, 2020.

[B17] Gordon C. J. Tchesnokov E. P. Feng J. Y. Porter D. P. Gotte M (2020). The antiviral compound remdesivir potently inhibits RNA-dependent RNA polymerase from Middle East respiratory syndrome coronavirus. J. Biol. Chem. AC120. 013056. 10.1074/jbc.AC120.013056 PMC7152756 32094225

[B18] Grein J. Ohmagari N. Shin D. Diaz G. Asperges E. Castagna A. (2020). Compassionate Use of Remdesivir for Patients with Severe Covid-19. New Engl. J. Med. 10.1056/NEJMoa2007016 PMC7169476 32275812

[B19] Holshue M. L. DeBolt C. Lindquist S. Lofy K. H. Wiesman J. Bruce H. (2020). First case of 2019 novel coronavirus in the United States. New Engl. J. Med. 10.1056/NEJMoa2001191 PMC7092802 32004427

[B20] INSERM (2020). Trial of Treatments for COVID-19 in Hospitalized Adults (DisCoVeRy), Available at: https://clinicaltrials.gov/ct2/show/NCT04315948 Accessed on 20 March, 2020.

[B21] Kuiken T. Fouchier R. A. Schutten M. Rimmelzwaan G. F. Van Amerongen G. van Riel D. (2003). Newly discovered coronavirus as the primary cause of severe acute respiratory syndrome. Lancet 362, 263–270. 10.1016/S0140-6736(03)13967-0 12892955 PMC7112434

[B22] Lim Y. X. Ng Y. L. Tam J. P. Liu D. X. (2016). Human coronaviruses: a review of virus host interactions. Diseases 4, 26. 10.3390/diseases4030026 PMC5456285 28933406

[B23] NIAID (2020). Adaptive COVID-19 Treatment Trial, Available at: https://clinicaltrials.gov/ct2/show/NCT04280705 Accessed on 20 March, 2020.

[B24] Sheahan T. P. Sims A. C. Graham R. L. Menachery V. D. Gralinski L. E. Case J. B. (2017). Broad-spectrum antiviral GS-5734 inhibits both epidemic and zoonotic coronaviruses. Sci. Trans. Med. 9. 10.1126/scitranslmed.aal3653 PMC5567817 28659436

[B25] Sheahan T. P. Sims A. C. Leist S. R. Schafer A. Won J. Brown A. J. (2020). Comparative therapeutic efficacy of remdesivir and combination lopinavir, ritonavir, and interferon beta against MERS-CoV. Nat. Commun. 11, 1–14. 10.1038/s41467-019-13940-6 31924756 PMC6954302

[B26] Siegel D. Hui H. C. Doerffler E. Clarke M. O. Chun K. Zhang L. (2017). Discovery and Synthesis of a Phosphoramidate Prodrug of a Pyrrolo [2, 1-f][triazin-4-amino] Adenine C-Nucleoside (GS-5734) for the Treatment of Ebola and Emerging Viruses. J. Med. Chem. 60, 1648–1661. 10.1021/acs.jmedchem.6b01594 28124907

[B27] Tang W. Cao Z. Han M. Wang Z. Chen J. Sun W. (2020). Hydroxychloroquine in patients with COVID-19: an open-label, randomized, controlled trial. medRxiv. 10.1101/2020.04.10.20060558

[B28] US-AMRDC (2020). Expanded Access Remdesivir (RDV; GS-5734™)>, available at: https://clinicaltrials.gov/ct2/show/NCT04302766 Accessed on 20 March, 2020.

[B29] Wang M. Cao R. Zhang L. Yang X. Liu J. Xu M. (2020). Remdesivir and chloroquine effectively inhibit the recently emerged novel coronavirus, (2019-nCoV) in vitro. Cell Res. 30, 269–271. 10.1038/s41422-020-0282-0 32020029 PMC7054408

[B30] Wang Y. Zhang D. Du G. Du R. Zhao J. Jin Y. (2020). Remdesivir in adults with severe COVID-19: a randomised,double-blind, placebo-controlled, multicentre trial. Lancet. 10.1016/S0140-6736(20)31022-9 PMC7190303 32423584

[B31] Weiss S. R. Leibowitz J. L. (2011). Coronavirus pathogenesis Adv. Virus Res. 81, 85–164. 10.1016/B978-0-12-385885-6.00009-2. 22094080 PMC7149603

[B32] WHO (2020). WHO. World Health Organization situation report. Available at: https://www.who.int/docs/default-source/coronaviruse/situation-reports/20200316-sitrep-56-covid-19.pdf?sfvrsn=9fda7db2_2 Accessed on 20 March, 2020.

[B33] Yethindra V. (2020). Role of GS-5734 (Remdesivir) in inhibiting SARS-CoV and MERS-CoV: The expected role of GS-5734 (Remdesivir) in COVID-19 (2019-nCoV)-VYTR hypothesis. Int. J. Res. Pharm. Sci. 11, 1–6. 10.26452/ijrps.v11iSPL1.1973

[B34] Zhang L. Zhou R. (2020). Binding Mechanism of Remdesivir to SARS-CoV-2 RNA Dependent RNA Polymerase. Preprints. 202003026. 10.20944/preprints202003.0267.v1 32521159

[B35] Zhu N. Zhang D. Wang W. Li X. Yang B. Song J. (2020). A Novel Coronavirus from Patients with Pneumonia in China 2019. New Engl. J. Med. 10.1056/NEJMoa2001017 PMC7092803 31978945

